# Fetal growth restriction and stillbirth: Biomarkers for identifying at risk fetuses

**DOI:** 10.3389/fphys.2022.959750

**Published:** 2022-08-19

**Authors:** Victoria J. King, Laura Bennet, Peter R. Stone, Alys Clark, Alistair J. Gunn, Simerdeep K. Dhillon

**Affiliations:** ^1^ Fetal Physiology and Neuroscience Group, Department of Physiology, The University of Auckland, Auckland, New Zealand; ^2^ Department of Obstetrics and Gynaecology, The University of Auckland, Auckland, New Zealand; ^3^ Auckland Biomedical Engineering Institute, The University of Auckland, Auckland, New Zealand

**Keywords:** fetal growth restriction (FGR), stillbirth, fetal hypoxia, biomarkers, fetal heart rate variability (fHRV), preterm brain injury

## Abstract

Fetal growth restriction (FGR) is a major cause of stillbirth, prematurity and impaired neurodevelopment. Its etiology is multifactorial, but many cases are related to impaired placental development and dysfunction, with reduced nutrient and oxygen supply. The fetus has a remarkable ability to respond to hypoxic challenges and mounts protective adaptations to match growth to reduced nutrient availability. However, with progressive placental dysfunction, chronic hypoxia may progress to a level where fetus can no longer adapt, or there may be superimposed acute hypoxic events. Improving detection and effective monitoring of progression is critical for the management of complicated pregnancies to balance the risk of worsening fetal oxygen deprivation *in utero*, against the consequences of iatrogenic preterm birth. Current surveillance modalities include frequent fetal Doppler ultrasound, and fetal heart rate monitoring. However, nearly half of FGR cases are not detected *in utero*, and conventional surveillance does not prevent a high proportion of stillbirths. We review diagnostic challenges and limitations in current screening and monitoring practices and discuss potential ways to better identify FGR, and, critically, to identify the “tipping point” when a chronically hypoxic fetus is at risk of progressive acidosis and stillbirth.

## Introduction

Fetal growth restriction (FGR) is defined as a fetus that fails to reach its biologically determined growth potential (Blencowe et al., 2019; Biks et al., 2021). The global prevalence is suggested to be around 20.5% (Blencowe et al., 2019). The incidence is reported to be higher in low- and middle-income countries; although accurate data are lacking (Blencowe et al., 2019; Biks et al., 2021). FGR is strongly associated with adverse pregnancy outcomes including stillbirth, preterm birth, increased neonatal mortality, increased risk for adult diseases, and neurodevelopmental disability (Malhotra et al., 2019; Fung and Zinkhan, 2021; Dudink et al., 2022). In principle, accurately identifying and managing FGR cases should be central to reducing mortality and morbidity. In practice, currently more than 50% of cases of FGR are undetected even in high income countries (McCowan et al., 2018; Malhotra et al., 2019), and more than 70% of babies with FGR who die antepartum are not diagnosed (Pacora et al., 2021). Further, even when FGR is correctly identified, there are only limited tools to monitor the severity of fetal oxygen deprivation, and thereby attempt to balance the risks of stillbirth or impaired development against the risks of premature delivery (Damhuis et al., 2021; Lausman and Kingdom, 2021). In this review, we will discuss the diagnostic challenges for FGR, the limitations of available monitoring techniques, and potential biomarkers that may help to improve detection and management of FGR.

## Small for gestational age vs. fetal growth restriction: Definitions

One of the key difficulties in developing biomarkers to better identify FGR is distinguishing those who are truly growth restricted due to pathological circumstances (e.g. oxygen and nutrient deprivation) from those who are small for gestational age (SGA), but have normal placental gas exchange or are healthy (Khalil et al., 2015; Damhuis et al., 2021; Lees et al., 2022). This is a major problem for research, since if a biomarker discovery cohort included a mixed group of infants with SGA, only some of whom had FGR, the inconsistency in definition would confound any association.

SGA fetuses are typically defined by an estimated birth weight below the 10th percentile or less than two standard deviations below the mean for their age, sex and parity. There is growing evidence that birth weight standards customized to specific populations, and incorporating factors such as ethnicity and maternal height and body mass index, can improve identification of FGR at preterm gestational ages (Anderson et al., 2016; Gardosi et al., 2018; Damhuis et al., 2021; Lees et al., 2022). SGA and FGR are not synonymous; in practice, FGR is an overlapping subset of SGA (Gordijn et al., 2016; Damhuis et al., 2021; Deter et al., 2021). Most infants with early-onset FGR are SGA, though around 40% of babies <10th percentile are constitutionally small and healthy (Khalil et al., 2015).

Conversely, late-onset FGR with placental insufficiency may lead to restricted fetal growth compared to their growth potential, but with a birth weight in the normal range (i.e. be AGA) (Gordijn et al., 2016; Damhuis et al., 2021; Deter et al., 2021). Up to 70% of deaths before birth occur in fetuses who were considered to be AGA (Pacora et al., 2021). Heterogeneity of growth throughout gestation further complicates accurate diagnosis, highlighting the value of individualized longitudinal assessments (Deter et al., 2021).

These data tell us that although fetuses whose weight is < 3rd percentile have significantly higher risks of adverse events, fetal size alone cannot be sufficient to accurately predict FGR and its associated risks (Unterscheider et al., 2013). It is now recognized that accurately identifying FGR and thus risk requires a broader suite of measures as proposed by the Delphi procedure consensus criteria (Gordijn et al., 2016; Beune et al., 2018). In addition to redefining estimated fetal weight to better stratify risk for very small (weight <3rd percentile) and small (>3rd to <10th percentile) babies and assessing maternal comorbidities, further assessment variables include placental, umbilical and fetal blood flow, fetal heart rate analysis, gestational age, assessment of the fetal biophysical profile and longitudinal growth trajectory and diagnostic and/or prognostic biochemistry (Gordijn et al., 2016; Beune et al., 2018). Numerous studies are now assessing and validating these measurements, adding to the data obtained from other recent assessment trials such as the Growth Restriction Intervention Study (GRIT) and The Trial of Umbilical and Fetal Flow in Europe (TRUFFLE) (Khalil et al., 2019; Ganzevoort et al., 2020; Lausman and Kingdom, 2021).

It is important to appreciate that FGR is a biological continuum. The timing of FGR is an important variable. Around 20–30% of FGR cases are early-onset (onset <32 weeks gestation). These fetuses have much greater risk of mortality and morbidity (Figueras and Gratacós, 2014; Mifsud and Sebire, 2014; Dall’Asta et al., 2017; Baschat, 2018; Lees et al., 2022). Late-FGR (≥32 weeks) is still associated with risk of adverse perinatal events and outcomes, including late preterm birth, sudden fetal deterioration and hypoxia and stillbirth (Baschat, 2018; Figueras et al., 2018).

## Etiology

The etiology of FGR is multifactorial, and can include impaired placental development and function, pre-eclampsia and maternal hypertension, and poor maternal cardiovascular adaptation to pregnancy, multiple gestation, maternal diabetes, maternal environment (e.g. high altitude, asthma, and stress) and lifestyle factors such as smoking, drug-use, malnutrition, and placental and fetal abnormalities (genetic or otherwise) (Sharma et al., 2016; Burton and Jauniaux, 2018; Damhuis et al., 2021; Nowakowska et al., 2021). Infections such as cytomegalovirus, syphilis and hepatitis C can also be associated with FGR (Longo et al., 2014). SARS-Cov2 infection may cause FGR secondary to the formation of placental lesions and placental insufficiency (Dubucs et al., 2022).

Ultimately, the most common underlying pathogenesis involves impaired placental development and function, leading to reduced supply of nutrients and oxygen (Mifsud and Sebire, 2014; Burton and Jauniaux, 2018; Zur et al., 2020). Maternal vascular malperfusion is the most common placental cause linked with FGR. It can arise from a variety of placental abnormalities, which can be interconnected, from placental hypoplasia to infarcts and lesions (Zur et al., 2020). Failure of extravillous cytotrophoblast invasion leading to poor placental structure and inadequate remodeling of spiral arteries (the maternal arteries that directly supply the placenta), for example, impairs perfusion (Burton and Jauniaux, 2018), giving rise to altered vascular resistance, intra-placental vascular lesions and reduced surface area for maternal-fetal exchange (Mifsud and Sebire, 2014). Further, impaired placentation is associated with secondary factors that alter placental perfusion such as maternal hypertension and pre-eclampsia (Zur et al., 2020). While small, dysfunctional placentae are at the root of early-onset FGR and more severe FGR, impaired placental structure and function also underpins late-onset FGR. The difference is a matter of degree rather than different causal mechanisms *per se* (Figueras et al., 2018). Late-onset FGR may be associated with an increased incidence of fibrosis and reduced vascularity (Kovo et al., 2010; Mifsud and Sebire, 2014; Figueras et al., 2018).

## Fetal adaptation to growth restriction–vulnerability to further hypoxia?

The fetus can detect and respond to hypoxic challenges to its homeostasis from very early in gestation (0.3 gestation, as seen in fetal sheep), and makes adjustments to match growth with energy availability if the hypoxia persists and the fetus is able to adapt appropriately (Kiserud et al., 2001; Giussani, 2016; Bennet, 2017; Lear et al., 2018).

Early-onset FGR is usually characterized by symmetrical growth, where reduced growth of the head and body are in proportion. By contrast, later-onset FGR is typically characterized by asymmetrical growth, where the abdominal circumference is reduced <10th percentile, while other measurements (especially head circumference) are relatively preserved and may be within normal limits (Figueras et al., 2018). Asymmetrical growth is thus associated with so-called “brain sparing,” with redistribution of blood flow away from the periphery to central beds to support organs such as the brain (Kiserud et al., 2001; Giussani, 2016; Bennet, 2017; Lear et al., 2018). The word “sparing” should not, however, be taken to mean normal brain growth in all cases, given that brain injury and impaired brain development are observed in even fetuses with asymmetrical FGR (Miller et al., 2016; Dudink et al., 2022). Indeed, brain sparing is not associated with improved executive function and behavioral outcomes or cognitive function in school-age children (Benítez-Marín et al., 2021). Neurological sequelae undoubtedly reflect that FGR is a continuum, and the specific outcomes must reflect the timing of placental insufficiency (i.e. stage of neural development) as well as severity of the challenge (Miller et al., 2016; Malhotra et al., 2019; Dudink et al., 2022).

### Insights from animal models

Critically, pre-clinical studies in a variety of species demonstrate that FGR is also associated with effects on other organs such as the kidney, with reduced nephron endowment and associated reduced filtration capacity (Mitchell et al., 2004), and heart, with reduced cardiac myocyte endowment, impaired maturation, remodeling of cardiac vasculature and a switch in energy substrate use (Louey et al., 2007; Wang et al., 2013; Botting et al., 2018; Masoumy et al., 2018; Maréchal et al., 2021; Drake et al., 2022). Altered renal and cardiac development are strongly linked with later-life risks for cardiovascular disease (Masoumy et al., 2018; Fung and Zinkhan, 2021). However, altered cardiac maturation and energy management may also affect fetal survival during further episodes of hypoxia. For example, a recent study evaluating cardiac metabolism in growth retarded fetal sheep using two imaging modalities, two-photon microscopy and phase-contrast magnetic resonance imaging, found evidence that the FGR heart relies heavily on glycolysis for ATP production, consistent with an impaired ability to tolerate further hypoxia (Dimasi et al., 2021). We have previously shown that fetal weight was strongly associated with impaired tolerance to severe hypoxia in preterm fetal sheep, particularly in males, and that the pattern of impaired failure of tolerance to hypoxia was sex dependent (Bennet et al., 2007). Others report that FGR reduces cardiac myocyte endowment in a sex-specific manner (Botting et al., 2018) and alters cardiac energy management (Maréchal et al., 2021).

Hypoxia secondary to placental dysfunction is a key feature of stillbirth, both as a direct feature of placental dysfunction and because it is associated with exacerbation of fetal compromise during secondary insults. Thus, it is vital to understand whether fetuses with FGR have compromised ability to respond to further hypoxic challenges (Pacora et al., 2019). The fetal chemoreflex and other defensive adaptations to hypoxia and more severe insults such as asphyxia are well described (for reviews *see* (Giussani, 2016; Lear et al., 2016; Bennet, 2017)). In chronically instrumented fetal sheep, the pattern of fetal adaptation to severe hypoxic challenge is similar at all gestational ages, with similar stages of compensation before final decompensation, but that younger fetuses are able to survive severe hypoxia for much longer than older fetuses, consistent with their known anaerobic tolerance and high levels of cardiac glycogen (Dawes et al., 1959; Shelley, 1964; Bennet, 2017). Unfortunately, surviving and thriving are very different, and to date, there is surprisingly limited data on the responses of the FGR fetus to superimposed episodes of acute hypoxia.

Acute hypoxia superimposed on chronic hypoxia may contribute to some antenatal and intrapartum fetal deaths (Pacora et al., 2019). For others, there may simply come a point when placental reserve fails and chronic hypoxia transitions to effectively represent acute severe hypoxia. Under such conditions the fetus must make further defensive responses, which include further redistribution of blood flow to vital organs, and conservation of energy through reduced body and breathing movements and other fetal activity (Pagani et al., 2014; Binder et al., 2018). Reduced fetal movements are strongly associated with fetal death (Heazell et al., 2018a; Ter Kuile et al., 2021). Further, abrupt fetal movements have been observed before stillbirth (Heazell et al., 2018b; Thompson et al., 2021). It has been speculated that this may be related to fetal seizures (Heazell et al., 2018b; Whitehead et al., 2020). Alternatively though, in fetal sheep, abrupt movements are seen shortly after the onset of an acute asphyxial insult (Bennet, 2017).

Studies in fetal sheep models have examined the impact of chronic hypoxia on the fetus’ ability to respond to acute insults, although many questions remain. In healthy near-term fetal sheep, brief umbilical cord occlusions repeated at a frequency that is consistent with early labor (1 min of occlusion every 5 min) can be fully compensated for, with no hypotension and only mild acidosis (Bennet et al., 2005). Near-term fetal sheep with pre-existing hypoxia of unknown origin and duration exposed to the same challenge show enhanced initial chemoreflex response to hypoxia (Bennet et al., 2005) and earlier centralization of blood flow (Wibbens et al., 2007). However, these early defense responses are not maintained; fetuses with pre-existing hypoxia become metabolically acidotic and hypotensive during repeated umbilical cord occlusion compared with healthy fetuses, and experience more severe decompensation (Westgate et al., 2005; Pulgar et al., 2006; Wassink et al., 2013; Amaya et al., 2016). These data suggest that autonomic responses to hypoxia are intact in FGR, and in some respects enhanced, but anaerobic reserves such as cardiac glycogen are insufficient to maintain cardiovascular adaptation.

There is some evidence of altered responses from preclinical studies in induced FGR. In a classic study, embolization of the placental circulation of late gestation (0.75 gestation) fetal sheep for ∼2 weeks induced moderate hypoxia without acidosis, resulting in a 31% reduction in fetal growth, and redistribution of combined ventricular output with an increased brain-liver ratio (asymmetrical growth) (Block et al., 1989). The fetuses were then challenged with a short period of isocapnic hypoxia induced by reduction of the maternal FiO_2_ to ∼10%. The FGR fetuses mounted a greater circulatory defense response than control fetuses, but without a change in umbilical blood flow. The authors queried if this could be sustained, and if better early compensation would be followed by quicker decompensation. A study of only 9 days embolization producing a 20% reduction in fetal growth and similar hypoxia, showed no redistribution of blood flow prior to the acute hypoxic challenge (symmetrical growth), and no differences in the circulatory response to acute hypoxia between FGR and control groups (Block et al., 1990). The authors concluded that under these circumstances the FGR fetuses were able to mount a comparable cardiovascular defense to hypoxia, and would do so until the placental reserve was completely depleted.

An alternative approach is to induce growth restricted pregnancy by removal of placental sites pre-implantation (carunculectomy) (Robinson et al., 1983). In this study, fetal weight was highly variable, ranging from no change to a 60% reduction compared with controls, but all fetuses were hypoxic without acidosis. In response to superimposed isocapnic hypoxia, FGR fetuses initially responded with acute bradycardia, similarly to controls, but interestingly, heart rate returned to baseline within a short period. This likely reflects higher circulating levels of catecholamines as reported in this model (Llanos et al., 1980; Walker et al., 1990). The increased cardiac work due to higher heart rates would likely deplete cardiac glycogen, contributing to earlier decompensation (Bennet, 2017). Blood pressure was slower to rise in the FGR group compared to controls, suggesting altered peripheral vascular resistance.

Consistent with this, chronically hypoxic fetal sheep raised at altitude and exposed to an acute episode of isocapnic hypoxia had a reduced capacity to induce peripheral vasoconstriction in order to maintain centralization of blood flow (Herrera et al., 2016). In this study, chronically hypoxic fetuses did not exhibit classical changes in heart rate, blood pressure or peripheral blood flow during superimposed hypoxia. This might be due to impaired chemoreflex defenses or more simply to an altered homeostatic set point, given that the relative change from basal oxygenation was smaller in the FGR group. Similar high-altitude models of FGR have found that early-onset chronic fetal hypoxia alters vascular structure, vasoreactivity and cardiac function (Pulgar et al., 2009; Ducsay et al., 2018). The impact of altered vascular function was also noted in a late-onset FGR model induced by sheep living in an isocapnic hypoxia chamber from ∼121 days of gestation for 10 days (Allison et al., 2020). The chronically hypoxic fetuses exposed to acute hypotension demonstrated an impaired ability to restore blood pressure homeostasis, due to an absent baroreflex increase in fetal heart rate and altered peripheral vascular sensitivity to adrenergic stimulation. An earlier fetal sheep study of prolonged partial cord compression between 125 and 128 days of gestation, followed by episodes of acute hypoxemia, suggests the fetal response relies less on peripheral vasoconstriction and depends more on umbilical vasodilation in order to maintain perfusion to essential beds (Gardner and Giussani, 2003).

These studies represent different paradigms of FGR, with different degrees and duration of hypoxia. It should further be noted that while an acute moderate isocapnic challenge allows assessment of the fetal chemoreflex, it does not represent a real-life hypoxia challenge. Overall, combined with the studies of spontaneous hypoxia discussed above, these studies suggest that FGR per se does not impair the initial chemoreflex defense responses to hypoxia, but that these critical responses may not be sustained in many fetuses, leading to earlier and potentially terminal decompensation. Clinically, the difficulty detecting FGR, particularly later-onset FGR, impedes studies of the impact of birth asphyxia in FGR. Moreover, 97% of deliveries in the TRUFFLE study of early-onset FGR were by Cesarean section (Lees et al., 2013), and so precluded labor-induced asphyxia. There are few clinical data regarding the effects of comorbid FGR on birth asphyxia.

**FIGURE 1:**
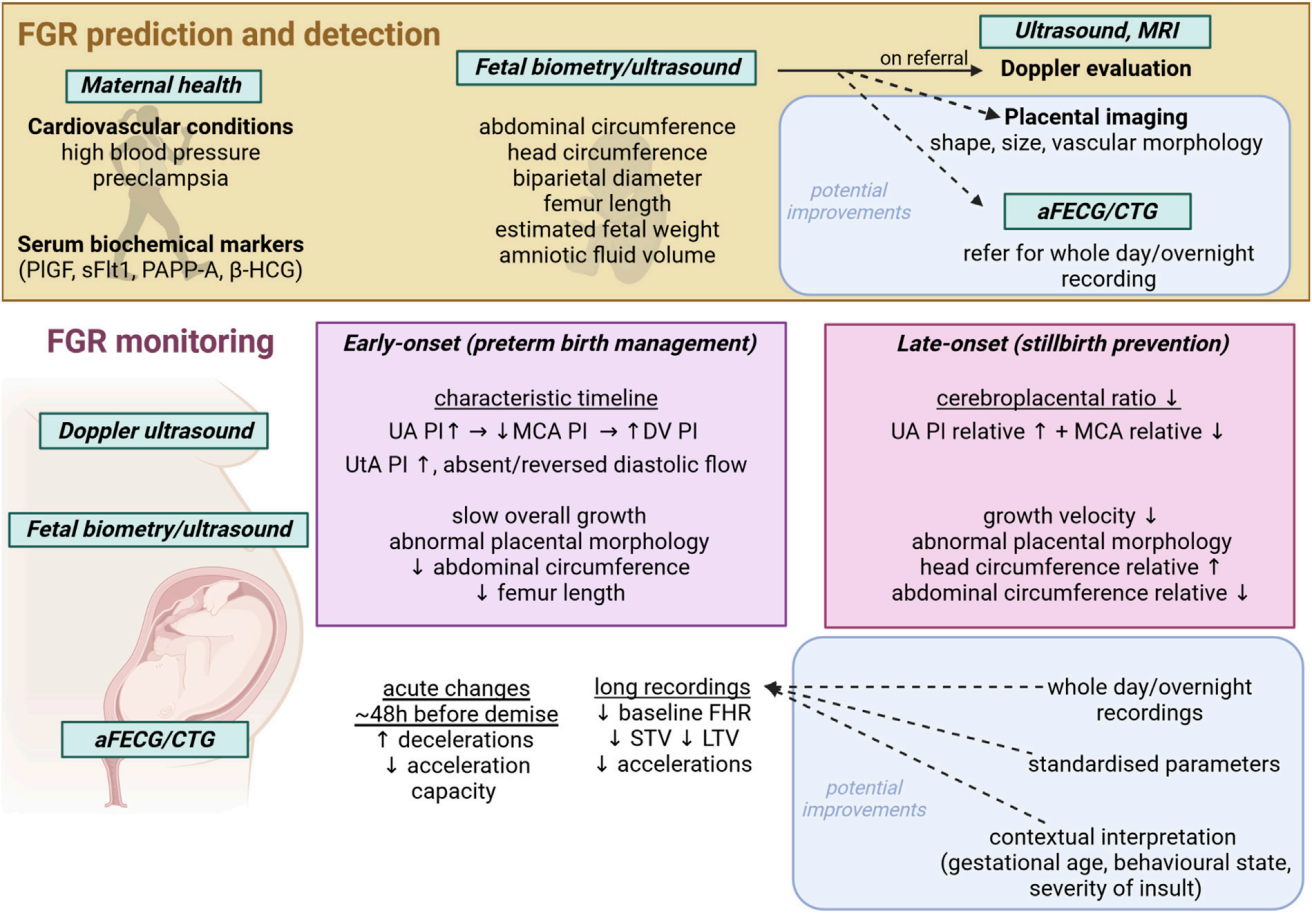
Schematic showing current FGR detection and monitoring techniques and modalities, with potential avenues for improvement. aFECG, abdominal fetal electrocardiogram; CTG, cardiotocogram; MRI, magnetic resonance imaging; UA, umbilical artery; MCA, middle cerebral artery; DV, ductus venosus; PI, pulsatility index; STV, short term variability; LTV, long term variability; PlGF, placental growth factor; sFlt1, soluble fms-like tyrosine kinase-1; PAPP-A, pregnancy associated plasma protein A; β-HCG, beta human chorionic gonadotropin; SPINT, serine protease inhibitor.

Thus, timely detection of FGR and at-risk fetuses is vital to reduce adverse outcomes (Baschat, 2018; McCowan et al., 2018; Ego et al., 2020). Detection of FGR alone can reduce the risk of stillbirth by nearly 40% as observed in a study of 92,182 births at ≥22  weeks of gestation (Ego et al., 2020). Effective monitoring of FGR progression is critical to balance the risk of worsening fetal oxygen deprivation and stillbirth against the risks of neonatal complications after preterm birth if the infant is delivered (Baschat, 2018; Delnord and Zeitlin, 2019). To improve outcomes, we need reliable biomarkers to identify FGR and to monitor evolving risks of deteriorating oxygenation or impact on neural development. Figure 1 summarizes current detection and monitoring techniques. Below, we briefly discuss current monitoring techniques, and advances in imaging and fetal heart rate monitoring.

## Doppler ultrasound, fetal biometry and biochemistry measures

Doppler ultrasound is routinely used to assess the hemodynamic aspects of placental dysfunction, as reviewed in detail (Vollgraff Heidweiller-Schreurs et al., 2018; Smith et al., 2021; Dall'Asta et al., 2022). Early-onset FGR is associated with abnormal umbilical and uterine vessel Doppler findings (Mifsud and Sebire, 2014; Baschat, 2018; Figueras et al., 2018; Lees et al., 2022) (*see* Table 1). In this setting, the umbilical artery (UA) waveform shows an increased pulsatility index (PI), and absent or reversed end diastolic velocities that can worsen as pregnancy progresses with increasing placental flow resistance (Cohen et al., 2016; Baschat, 2018; Caradeux et al., 2018; Morales-Roselló et al., 2018). The rate of increase in placental blood flow resistance is the primary determinant of early FGR progression and risk of fetal deterioration. A recent meta-analysis reported that the odds ratio of fetal death associated with early-onset FGR were 3.59 and 7.27 with absent and reversed UA end diastolic velocities, respectively (Caradeux et al., 2018). Furthermore, the extent of absent end diastolic velocity in the cardiac cycle was shown to be predictive of the risk of fetal death (Kinoshita et al., 2021). Similarly, absent and reversed ductus venosus flow are associated with worsening placental dysfunction and impaired fetal cardiac function. In early-onset FGR with absent or reversed end diastolic UA flow, abnormal ductus venosus Doppler measures were associated with further increased risk of stillbirth (Caradeux et al., 2018).

**TABLE 1:** Summary of select studies of potential biochemical and physiological markers of FGR.

Markers	Design	Subjects	Finding	Reference
Doppler ultrasound measures of UA, MCA, CPR	Systematic review and meta-analysis	31 studies (mix of observational cohort studies and RCTs of early-onset FGR (diagnosed <34 weeks)	Increased risk of death for early-onset FGR fetuses with absent or reversed end-diastolic velocities in either the UA (OR 3.59 absent, 7.27 reversed) or DV (OR 11.6, absent or reversed)	Caradeux et al. (2018)
	Systematic review and meta-analysis	128 studies (mix of prospective, retrospective; mix of CPR alone, MCA Doppler alone and both CPR and MCA Doppler)	CPR-PI outperforms UA and MCA Doppler in prediction of composite adverse outcome (0.59 sensitivity, 0.91 specificity) and emergency delivery for fetal distress (0.58 sensitivity, 0.89 specificity)	Vollgraff Heidweiller-Schreurs et al. (2018)
	Cohort analysis of two European multicenter trials (GRIT and TRUFFLE)	26–36 weeks gestation pregnant women, stratified by monitoring method for delivery	Early FGR monitoring with both cCTG and DV Doppler assessment was associated with a trend of improved survival without impairment at 2 years (84%), compared with only cCTG monitoring (80% GRIT; 77% TRUFFLE) or immediate delivery (70%)	Ganzevoort et al. (2020)
	Prospective RCT (TRUFFLE study)	26–32 weeks gestation singleton early-onset FGR pregnancy	Using late changes in DV waveform to inform delivery may improve 2-year outcomes	Lees et al. (2015)
	Delphi consensus	45 experienced clinical opinions	Early-onset FGR: UtA-PI and/or UA-PI >95th percentile	Gordijn et al. (2016)
			Late-onset FGR: CPR <5th percentile or UA-PI >95th percentile	
Maternal serum markers	Prospective case-control	15 control, 15 FGR pregnancies	Maternal serum proteome profiling: Proapolipoprotein C-II, apolipoprotein C-III1, and apolipoprotein C-III2 constitute IUGR signature (sensitivity 0.73, specificity 0.87, AUC 0.86)	Wölter et al. (2016)
Pregnancy-associated plasma protein A	Prospective	First trimester screening study in 786 pregnant women (3.2% SGA)	<5th percentile PAPP-A group (0.37 MoM) during first trimester associated with SGA (sensitivity 0.10, specificity 0.97, PPV 0.16, NPV 0.95)	Genc et al. (2022)
	Systematic review and meta-analysis	32 studies of first trimester screening in 175,240 pregnancies	<5th percentile PAPP-A group associated with birth weight <10th centile OR 2.08, <5th centile OR 2.83. Birthweight <5th centile LR +ve 2.65, LR −ve 0.85	Morris et al. (2017)
Micro-RNAs	Retrospective case-control	80 AGA, 80 FGR pregnancies	Combination of microRNA profile (miR-16-5p, miR-20a-5p, miR-145-5p, miR-146a-5p, miR-181a-5p, miR-342-3p, and miR-574-3p) during the first trimester detected FGR pregnancies (sensitivity 0.4268, specificity 0.95, cut off >0.6578 at 0.1 FPR)	Hromadnikova et al. (2022)
Placental growth factors	Prospective case-control	32 uncomplicated, 49 SGA and 126 FGR pregnancies	High ratio of placental growth factors (sFIt-1/PIGF) was associated with severity of early-onset FGR <97.4 stage I, up to 523.7 stage II, ≥523.7 stage III (PPV 0.986, 0.429, 0.462 respectively)	Garcia-Manau et al. (2021)
	Prospective observational	138 singleton pregnancies with EFW <10th centile between 20 and 31 weeks of gestation	sFIt-1/PIGF ratio cut-off value of 38 predictive of delivery before 2 weeks (NPV 1)	Bonacina et al. (2022)
	Secondary analysis of two RCTs	Preeclampsia Intervention with Esomeprazole trial (22 AGA infants BW > 10th centile and 75 SGA infants BW < 10th centile) and Preeclampsia Intervention 2 trial (40 AGA infants BW > 10th centile and 95 SGA infants BW < 10th centile)	SPINT1 was decreased in pre-eclamptic pregnancies complicated by growth restriction. Ratios of sFlt-1/SPINT1 and sFlt1/PlGF were increased	Murphy et al. (2022)
	Secondary analysis of two RCTs	Maternal samples were assessed from the fetal longitudinal assessment of growth 2 study (152 AGA and 75 SGA) and the biomarker and ultrasound measures for preventable stillbirth study (198 SGA 198, 23 preeclampsia cases and 182 controls)	At 36 weeks of gestation, circulating SPINT2 concentration was increased in patients who developed preeclampsia or delivered a SGA infant	Murphy et al. (2021)
Human chorionic gonadotropin	Retrospective	1900 AGA and 146 FGR+PE pregnancies	Second trimester intact hCG (>3 MoM) associated with increased risk of developing FGR	Sharony et al. (2018)
Midkine	Prospective case-control	72 AGA, 72 SGA pregnancies	High maternal serum Midkine at ∼36 weeks of gestation predictive of idiopathic FGR at term (sensitivity 0.63, specificity 0.64 at cut-off value 0.20)	Oluklu et al. (2022)
Maternal cardiovascular markers	Retrospective	136 AGA, 16 FGR pregnancies	High maternal peripheral vascular resistance (>1355) at 22–24 weeks gestation is predictive of FGR (sensitivity 0.842, specificity 0.932, AUC 0.88)	Vasapollo et al. (2022)
Placental MRI	Observational	12 AGA and 14 early-onset FGR pregnancies	FGR placenta have slow intervillous blood flow and patchy unperfused areas. Perfusion dynamics worsen with intermittent perturbations in flow	Brunelli et al. (2010)
	Observational	17 FGR and 36 normal pregnancies, between 28 and 38 weeks gestation	Placental perfusion fraction lower in FGR	Liu et al. (2021)
	Prospective observational	79 control 35 FGR pregnancies between 18 and 39 weeks gestation	Placental volumes smaller in FGR vs. controls	Andescavage et al. (2017)
	Prospective observational	94 control, 36 FGR/SGA pregnancies >18 weeks gestation	Microstructural alterations in FGR, particularly late-onset FGR	Andescavage et al. (2020)
	Prospective observational	46 controls, 34 FGR pregnancies between 18 and39 weeks gestation	Proposed placental volume algorithm can identify FGR (0.86 accuracy, 0.77 precision, 0.86 recall, AUC 0.86)	Dahdouh et al. (2018)
	Retrospective case control	1163 SGA (birthweight <3rd percentile) and 1163 sex and gestational age matched controls	LTV and STV in FHR have better predictive accuracy earlier (<34 weeks) in gestation. Marker values vary with fetal behavioral state	Stroux et al. (2017)
	Two separate prospective studies	singleton pregnancy; 31 SGA (EFW <10th percentile for gestational age) and 30 AGA controls	SGA does not show differences in Dawes and Redman parameter set between day and night; AGA does	Kapaya et al. (2018)
	Retrospective cross-sectional study	9071 normal, 1986 SGA (birthweight <10th percentile), 543 extreme SGA (birthweight <3rd percentile)	SGA fetuses have lower baseline heart rate from 34 weeks, lower STV and LTV, fewer accelerations compared with AGA fetuses	Amorim-Costa et al. (2017a)
	Prospective case control	66 SGA (abdominal circumference <5th percentile) and 79 uncomplicated pregnancies	Decrease of AC (OR 2.1) and DC (OR 0.5) in SGA fetuses from 25 weeks gestation compared with AGA; association is stronger in cases with brain-sparing (MCA-PI)	Stampalija et al. (2016)

DV, ductus venosus; UA, umbilical artery; MCA, middle cerebral artery; CPR, cerebroplacental ratio; PI, pulsatility index; sFlt1, soluble fms-like tyrosine kinase-1; PlGF, placental growth factor; PE, pre-eclampsia; EFW, estimated fetal weight; cCTG, computerized cardiotocography; MoM, multiple of the median; AUC, area under the curve.

The majority of cases of FGR are late-onset and more difficult to detect as they present with more subtle uterine or umbilical Doppler changes, or even with apparently normal flows, despite histological evidence of compromise in the placenta (Mifsud and Sebire, 2014; Baschat, 2018; Figueras et al., 2018; Dall'Asta et al., 2022; Lees et al., 2022). Redistribution of fetal combined ventricular output, with increased perfusion to the brain and associated brain-sparing, is a key indicator of the presence of fetal hypoxemia and adaptation. Thus, Doppler measurements of the middle cerebral artery (MCA) provide some utility in identifying at risk fetuses. Abnormal MCA Doppler measures in in later-onset FGR may, for example, be associated with increased risk of stillbirth within the next 4–7 days (Baschat, 2018). It is recognized that the difference between the pulsatility index of the UA and MCA (the cerebroplacental ratio, CPR) is more sensitive than either alone (Kalafat and Khalil, 2018; Vollgraff Heidweiller-Schreurs et al., 2018; Ciobanu et al., 2019). An abnormal CPR is associated with increased risk of stillbirth, and neonatal morbidity including adverse neurodevelopmental outcomes in survivors (Crimmins et al., 2014; Khalil and Thilaganathan, 2017; Kalafat et al., 2019; Monteith et al., 2019). It may also discriminate between SGA and FGR fetuses in cases of poor maternal vascular perfusion placental pathologies, but is reported to have limited utility for excluding FGR in other types of placental pathologies (i.e. fetal vascular malperfusion, or villitis of unknown etiology) (Ashwal et al., 2022).

However, like MCA measures, CPR alone remains a relatively poor predictor of perinatal outcomes in isolation, and combined measures are more predictive (Dall'Asta et al., 2019; D'Antonio et al., 2020; Schiffer et al., 2021; Vollgraff Heidweiller-Schreurs et al., 2021). There is a need for gestational age-based reference ranges for the pulsatility index from all vascular beds (Ciobanu et al., 2019). Fetal biometric information, such as ultrasound measures of abdominal circumference and femur length, are used to estimate fetal weight and provide additional information (Kiserud et al., 2017; Hiersch and Melamed, 2018). Some evidence suggests that universal third trimester ultrasound tripled SGA infant detection, and combining this approach with Doppler velocimetry measures identified SGA infants at high risk of morbidity (Sovio and Smith, 2018). Worryingly, other studies have shown that diagnosis of FGR by third trimester ultrasound did not materially improve perinatal outcomes (Henrichs et al., 2019; Bonnevier et al., 2022). Some of this discrepancy may be due to different gestational ages at screening (Roma et al., 2015), which highlights the importance of timing in the interpretation of biomarkers. Further, in many clinical settings misdiagnoses can continue due to inconsistencies in reference charts and reporting (Paoletti et al., 2021).

Doppler measures and biometry in tandem with plasma biomarker factors is proposed to increase sensitivity to distinguish FGR from SGA and AGA and those fetuses at greater risk of adverse events, thereby guiding clinical decision making around extra monitoring and early delivery (Wölter et al., 2016; Sovio et al., 2020). A combination approach incorporating maternal biochemical markers with fetoplacental ultrasound measures produces moderate improvements in FGR detection over either technique alone (Miranda et al., 2017), and may be able to distinguish between early- and later-onset phenotypes (Crovetto et al., 2016). However, the evidence is inconsistent (McCowan et al., 2017) and presently, use of maternal serum markers is not recommended for routine screening due to a lack of evidence (Heazell et al., 2015).

These tools have been used to directly investigate the concept that clinical FGR may impair fetal responses. For example, during an oxytocin challenge, fetuses with suspected FGR >36 weeks gestation, without evidence of brain-sparing (Fu and Olofsson, 2006), were able to increase cerebral perfusion during oxytocin, whereas fetuses with established brain sparing showed little change, suggesting impaired ability to respond to an acute challenge.

## Plasma and maternal cardiovascular and serum biomarkers

Placental factors are released into maternal circulation from the early stages of placentation, and some factors change with altered placental growth, function and impaired oxygenation and/or damage to the placenta (Araujo Junior et al., 2021). A recent metabolomic study demonstrated high predictive accuracy for FGR with a combination of 3-hydroxybutyric acid, glycine and phosphatidylcholine with acyl-alkyl residue C42 (Bahado-Singh et al., 2022). Lipoproteins in maternal serum may have discriminatory value for distinguishing FGR from AGA pregnancy (Wölter et al., 2016), aligning with evidence for a maternal component in altered lipid metabolism in FGR pregnancy (Paules et al., 2020). Several potential predictors of placental dysfunction have been identified during first and second trimesters, including pregnancy associated plasma protein A, β-human chorionic gonadotropin, tyrosine kinase-1 and 2, hypoxia-inducible factors 1–3, vascular endothelial growth factors, placental growth factor, angiopoietin-1 and -2, soluble fms-like tyrosine kinase-1, placental growth factor and serine protease inhibitor (Audette and Kingdom, 2018; Sun et al., 2020; Murphy et al., 2021; Smith, 2021; Bahado-Singh et al., 2022).

Table 1 provides a summary of biochemical biomarkers of placental dysfunction and FGR. Used alone, however, such markers have poor positive predictive value for FGR and poor outcome (Conde-Agudelo et al., 2013). Additionally, many studies examine a cross-section of fetuses at a single-time-point, which may miss later-onset FGR, and makes it difficult to assess the modulating effect of gestational age (Pecks et al., 2016). Large scale, longitudinal trials are required to determine validity and specificity of biomarkers for FGR and risk of adverse events.

Altered maternal hemodynamics play a key role in the pathophysiology of FGR. In particular, maternal hypertension and altered peripheral vascular resistance are the hallmarks of pre-eclampsia, which is strongly associated with FGR (Kametas et al., 2022). There is now interest in whether general measures of maternal hemodynamics can be used to discriminate between FGR and SGA and to determine fetal risks. Perry and others have shown that lower maternal heart rate and cardiac output, higher blood pressure and greater peripheral and uterine artery resistance may distinguish between SGA and FGR fetuses (Perry et al., 2020). Increased peripheral vascular resistance in mothers with chronic hypertension is highly predictive of birthweight and FGR by mid-pregnancy (Vasapollo et al., 2022), and lower cardiac output and increased systemic resistance was observed with FGR. These parameters were also a strong indicator of subsequent neonatal hospitalization (Di Pasquo et al., 2021). Reduced maternal perfusion can lead to lesions related to hypoxia. However, the presence of placental lesions *per se* is not predictive of FGR (Ferrazzi et al., 1999; Figueras et al., 2009). These studies have not tracked longitudinal changes in function, and so FGR characterized by reduced growth velocity rather than Dephi consensus criteria (Kennedy et al., 2020; Molina et al., 2020) could have been miscategorized as a control pregnancy. Secondary applications of algorithms for predicting early-onset pre-eclampsia have been tested for their capacity to predict SGA at birth (Mendoza et al., 2022). Overall, they have an overall poor predictive value (Crovetto et al., 2017), preventing current use in screening a “low-risk” population (Blitz et al., 2016).

**FIGURE 2:**
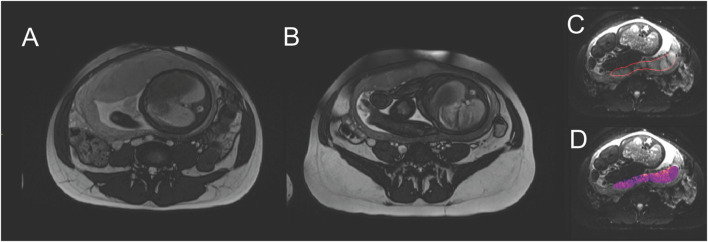
Example of placental MRI modalities. Comparing central slices through the placenta, **(A)** is a normal pregnancy (fetal abdominal diameter 96.5 mm) at 37 weeks gestation, with placental thickness of 41.2 mm. **(B)** is FGR (fetal abdominal diameter 79.2 mm, birthweight 1.4 centile) at 37 weeks gestation, with placental thickness of 21.8 mm. Placentae in FGR pregnancies are typically described as smaller and appearing darker and more heterogenous in MRI. **(C)** shows identification of a placental region of interest. **(D)** shows a parameter map of apparent diffusion coefficient, a parameter that relates to blood movement, and is typically extracted from MRI studies.

Given that increased vascular resistance and reduced fetoplacental blood flow are key factors in the mediating FGR, there is a need to better evaluate maternal and placental perfusion at the macro and micro level, using advanced approaches to ultrasound measurements and utilizing MRI, which allows more in-depth assessment of vascular structure, oxygenation and blood flow to provide insight into functionality (Aughwane et al., 2021; Schiffer et al., 2021; Clark et al., 2022a) (*see* Figure 2 for example). For instance, Clark and others have demonstrated that investigators need to evaluate the utero-placental vasculature as a whole, rather than just the spiral arteries, in order to better interpret uterine waveforms (Clark et al., 2018). Computational, biomechanical analysis may improve reliability of these measures (Clark et al., 2022b). Substantial work is still needed to validate both advanced ultrasound and MRI techniques experimentally and clinically (Andescavage and Limperopoulos, 2021; Saini et al., 2021; Clark et al., 2022a). Current data have shown that multi-compartment MRI can be used to evaluate local blood volumes within the placenta and fetoplacental blood oxygenation, and may be able to detect differences between FGR and normal pregnancies (Aughwane et al., 2021; Jacquier and Salomon, 2021). MRI can detect changes in the placentas of severe FGR fetuses with abnormal Doppler velocimetry (Ingram et al., 2017; Aughwane et al., 2021) and T2* values (which reflect, in part, oxyhemoglobin) are reportedly lower only in FGR cases with abnormal Doppler and where there is histological evidence of maternal hypoperfusion (Sinding et al., 2016). Placental pathologies have been shown in women with chronic hypertension using combined placental MR examination (e.g. T2 weighted imaging, T2*, T1 mapping and diffusion imaging (Ho et al., 2021)), and defined assessment templates using combined MR measurements allow for discrimination of normal and abnormal placental development (Ho et al., 2022). Diffusion weighted imaging has shown reduced placental perfusion in FGR pregnancies; Intravoxel Incoherent Motion (IVIM) models applied to MRI allow quantification of microstructure and blood flow without using contrast agents, and studies have shown utility in determining micro-vascularization changes in FGR pregnancies (Liu et al., 2021; Antonelli et al., 2022) without any sign of Doppler ultrasound impairment (Antonelli et al., 2022). Gestational age-associated changes in placental IVIM parameters likely reveal trajectories of microvascular perfusion fraction and diffusion characteristics in the developing placenta.

While MRI can provide valuable insights into fetal and placental disease progression in FGR (Kingdom et al., 2018), it is not particularly suitable as a screening technique as it demands intensive resources. Thus, we continue to seek other more generic monitoring modalities, that can be used serially and without expensive or intensive resources. In this regard, the final part of our review looks at antenatal fetal cardiotocography (CTG) used to derive fetal heart rate and other indices.

## Antenatal fetal heart rate and heart rate variability markers

In current practice, early-onset FGR is assessed with Doppler ultrasound and FHR variability (FHRV) measurements. Antenatal cardiotocography (CTG) involves monitoring fetal heart rate and uterine contractions by ultrasound. Changes in FHR reflect changes in fetal oxygen level, autonomic nervous system function, cardiovascular maturation and neurodevelopment, as behavioral state (the emergence of which is a key neurodevelopmental milestone) (Hoyer et al., 2017). Overt FHR abnormalities are largely only observed during a very short terminal phase. This may be preceded by a gradual reduction in FHRV, but in practice, marked suppression of short-term variability (STV) is only reliably seen in the terminal stage of FGR in the final 1–2 days before fetal demise or appropriate delivery (Baschat et al., 2022). At this terminal stage, suppressed STV may also be associated with FHR decelerations. This combination was observed in 44% of cases just before delivery in the Trial of Umbilical and Fetal Flow in Europe (TRUFFLE) study (Lees et al., 2015). This is consistent with the hypothesis that this pattern represents the terminal stage of failing uteroplacental function. Thus, current methods are both highly stressful to the family, as they require daily monitoring, and yet are still associated with only 77%–85% survival without neurodevelopmental impairment (Baschat et al., 2022).

### Improving FHR monitoring for FGR detection and monitoring

The time period and timing of recording is highly important in the interpretation of FHRV. This is due to varying fetal behavioral states. Before 32 weeks gestation, fetuses show cycles of quiescence and activity (Keen et al., 2011). In preterm life, fetal activity, such as breathing-like movements, body movements, sucking, yawning, swallowing is nearly continuous. From around 32 weeks gestation, distinct behavioral sleep states that were first identified in newborns and then detected by FHR in the fetus, begin to emerge (Keen et al., 2011). With maturation, the fetus spends about two-thirds of the time in active sleep (2F, characterized by a steady FHR, eye movements, and frequent body movements), a quarter in quiet sleep (1F, characterized by slow, steady FHR and few body movements) and the remainder in 4F (so-called “active awake,” characterized by fast, irregular FHR and continual eye and body movements) (Brändle et al., 2015). The reader should note that despite the terminology there is no evidence that the fetus is actually awake in any of these states (Mellor et al., 2005).

Preterm fetal sheep show a discontinuous EEG, containing a mixture of frequencies and amplitudes. As the fetus develops towards term, the EEG becomes continuous, with distinct low-frequency-high-amplitude (REM) and high-frequency-low amplitude (NREM) sleep states (Dawes et al., 1972; Richardson et al., 1994). REM states contain breathing movements and eye movements; NREM additionally has body movements (Szeto and Hinman, 1985). These EEG and behavioral patterns and their development are similarly seen in monitored preterm-born and term-born humans (Sandoval Karamian and Wusthoff, 2021) (for review, *see* (Bennet et al., 2018)).

Thus, FHR and derived indices can vary considerably depending on fetal behavioral state (Signorini et al., 2003). Therefore, differences in fetal sleep-state during recording can confound comparisons of FHRV measurements (Sriram et al., 2013). Prolonged fetal FHR recording would be required to capture both active and quiet sleep, which would require recording for 2 h or longer (Ryo et al., 2018; Sumiyoshi et al., 2020), and a calculated average may be used for comparison (Signorini et al., 2020). Additionally, sleep state development is altered with fetal hypoxemia (Boddy et al., 1974; Lee et al., 2009). Consistent with the impaired neurodevelopment of the chronically hypoxic fetus, in daily FHR recordings which began between 24 and 28 weeks gestation, SGA fetuses (EFW <10th percentile) were found to have a delay in FHR rhythm formation, indicative of delayed fetal behavioral state differentiation/sleep-state cycling formation, of around 1–2 weeks (Sumiyoshi et al., 2020). Further, a reduction in fetal movements is associated with fetal hypoxemia and FGR (Heazell and Frøen, 2008). Placental dysfunction and insufficient fetal oxygenation have been linked with reduced fetal movements (Ter Kuile et al., 2021), which is thought to be an oxygen conservation strategy (Warrander and Heazell, 2011). A combined assessment of FHRV with sleep-state pattern (derived as an index of fetal activity from the presence of accelerations and long term variability measures) can improve detection of FGR (Stroux et al., 2017).

Similarly, there are known circadian rhythms of FHR and variability; therefore, the timing of measurement may be important (Kapaya et al., 2016). Fetuses receive circadian cues from their mothers and have circadian rhythms in both physiological and pathophysiological conditions. Circadian rhythms in FHR have been observed as early as 20 weeks of gestation in human pregnancies (de Vries et al., 1987). FHR is known to increase in the late afternoon, peaking in the early-mid evening; in part, these measures reflect fetal behavior and the circadian timing of fetal movements (Bradford et al., 2019). Despite its limited predictive value, intermittent auscultation is standard practice to monitor FHR (Henderson et al., 2000; Haws et al., 2009; Murray, 2017). Clinical FHR monitoring is often performed indirectly using Doppler-based techniques; this necessarily limits monitoring time length and indeed, timing of monitoring, with sessions lasting 20–40 min (Murray, 2017). More frequent monitoring is performed only where clinical concern is identified, and sessions are relatively short, far less than a whole circadian period. The growth and circadian rhythmicity of normally developing fetuses is sufficient to produce conflicting results when measurements are taken day-on-day, and within a day measurements vary substantially, reflecting changing fetal behaviors (Lunshof et al., 1998; Babazadeh et al., 2005; Avitan et al., 2018).

Short duration recordings during prenatal visits do not capture sufficient data for these analyses (Ryo et al., 2018). Therefore, using an ECG for overnight fetal monitoring has gained interest for monitoring of the fetus. A study of FHR recordings for 20 h from singleton pregnancies (30 AGA and 31 SGA) reported that SGA fetuses had a blunted diurnal rhythm in STV, LTV, accelerations and time spent in high frequency activity, although the absolute values were within normal range (Kapaya et al., 2018). These findings suggest that the time of day can affect the interpretation of FHR and FHRV measures, and further studies are required to assess the diagnostic potential of diurnal change in FHR measures to identify FGR.

In addition to changes with fetal sleep-state and circadian pattern, there are maturational changes in FHR and FHRV over the course of gestation. Surprisingly, the majority of studies of CTG parameters in FGR fetuses did not control for gestational age, despite including fetuses from a range of gestation ages. A longitudinal study of 176 pregnancies (31 SGA) using sequential CTG recordings from 24 to 39 weeks of gestation reported that with advancing gestation age both SGA and AGA fetuses had a reduction in baseline FHR and increase in short-term and long-term HRV (Amorim-Costa et al.). However, SGA fetuses had a much steeper descent in baseline FHR and lower LTV than AGA (Amorim-Costa et al., 2017b). Similarly, a cross-sectional CTG study in a large cohort (9701 AGA and 1986 SGA (birth weight <10th percentile for gestational age)) reported that SGA fetuses had a lower baseline heart rate from 34 weeks of gestation, and lower average STV and LTV and fewer accelerations compared with AGA fetuses (Amorim-Costa et al., 2017a). These longitudinal differences in FGR may reflect relatively greater parasympathetic influence (Vinkesteijn et al., 2004; Shaw et al., 2018). Future studies assessing sequential changes in FHR and FHRV parameters in conjunction with fetal Doppler measures over gestation, and correlating these with fetal outcomes, will help to evaluate if these markers can be used for detection and management of FGR fetuses.

FGR fetuses are generally believed to be able to compensate for stable chronic hypoxemia-, and so they may have reduced physiological reserves to withstand additional insults as discussed earlier. Maternal supine sleep position is associated with fetal growth restriction and is an independent risk factor for late gestation stillbirth (Gordon et al., 2015). Notably, maternal obstructive sleep apnea has been associated with reduced fetal growth velocity (Fung et al., 2013; Kneitel et al., 2018). Sleep position can also alter maternal hemodynamics. A study using MRI in women in the third trimester of pregnancy found that the maternal supine position is associated with a 32% reduction in aorta flow and 85% reduction in flow through the inferior vena cava, suggesting increased reliance on collateral venous circulation to maintain cardiac output (Humphries et al., 2018). Moreover, maternal sleep position was associated with changes in fetal behavioral state and FHR variability (Stone et al., 2017). A study of CTG with maternal polysomnography in late pregnancy reported that nocturnal FHR decelerations and prolonged reduction in FHRV were more likely in pregnancies complicated by hypertension and/or FGR, and these changes were associated with maternal sleep position (Wilson et al., 2022). This suggests that an adverse event (e.g. a single apnea episode, or single position change) may initiate/provoke a hypoxic challenge, the response to which may be altered depending on extant fetal capacity and autonomic maturation (Robertson et al., 2019). Overnight observations might provide insight into the extent of fetal (de)compensation in FGR.

In FGR with brain-sparing, accelerative and decelerative capacity (AC and DC) are reduced from 25 weeks gestation to term, compared with normal fetuses (Stampalija et al., 2016; Frasch et al., 2018). In prenatal ambulatory CTG recordings (mean length 42 min) AC was able to distinguish between normal and FGR pregnancies, as was STV (Huhn et al., 2011). An observational study of overnight FHR recordings in normal and SGA pregnancies, STV, found that average AC and average DC were significantly lower in SGA pregnancy, though these parameters were unable to distinguish between the heterogeneous etiologies in the SGA group (Graatsma et al., 2012). While longer recordings are desirable for capture of relevant behavioral states in later gestation, even short recordings may have some value earlier in gestation. A retrospective analysis of 3min segments of FHR recordings taken from 28 weeks gestation onwards found that time-domain measures were able to discriminate between normal and FGR fetuses, prior to 36 weeks gestation (Esposito et al., 2019). It may be that such measures would be of most use before 34 weeks gestation (Stampalija et al., 2015; Tagliaferri et al., 2015). However, the recordings analyzed here are usually taken in the context of pre-selected groups, limiting the application of the findings with a view to create a screening tool. It is likely that a combinatorial approach to screening may offer the best predictive capacity to identify at-risk fetuses; for example, a study found that the combination of Doppler velocimetry and scheduled computerized CTG analysis was reasonably effective in preventing fetal demise in early-onset FGR (Wolf et al., 2020). Current efforts to generate a set of FHR-derived indices for screening purposes reiterate the need to incorporate factors such as gestational age and insult severity (Hoyer et al., 2019). Machine-learning approaches in addition to hypothesis-driven approaches are helping to identify and consolidate measures which might best discriminate the at-risk fetus (Signorini et al., 2020; Khatibi et al., 2021; Pini et al., 2021).

## Conclusion

Current screening techniques do not provide good detection rates for fetal growth restriction, and later-onset growth restriction in particular. Once detected, present monitoring strategies are not sufficient to provide timely and reliable detection of fetal compromise in FGR. There is urgent need to improve detection and monitoring of FGR to improve outcomes. Measures of fetal heart rate and variability are readily accessible and easily deployed in low-resource settings, but short-term recordings limit their utility. A thorough exploration of the efficacy of these measures requires research using longer, more frequent recordings. A better understanding of the utility of monitoring variation in FHR and derived indices with fetal behavioral state, circadian timing and maturation in FGR pregnancies is important. Advanced imaging techniques for assessing placental structure and function are also emerging as a promising tool for FGR detection. Preclinical studies with standardized animal models and computational modelling work will allow for deeper investigation of disease progression and biomarker identification. Once identified, suitable biomarker candidates could be incorporated in a combinatorial approach to a detection framework.
